# Transport and Recovery of Gilthead Sea Bream (*Sparus aurata* L.) Sedated With Clove Oil and MS222: Effects on Oxidative Stress Status

**DOI:** 10.3389/fphys.2019.00523

**Published:** 2019-05-03

**Authors:** Mariana Teles, Miguel Oliveira, Ismael Jerez-Cepa, Lorena Franco-Martínez, Asta Tvarijonaviciute, Lluis Tort, Juan M. Mancera

**Affiliations:** ^1^Department of Cell Biology, Physiology and Immunology, Universitat Autònoma de Barcelona, Barcelona, Spain; ^2^Department of Biology, Centre for Environmental and Marine Studies, University of Aveiro, Aveiro, Portugal; ^3^Departamento de Biología, Facultad de Ciencias del Mar y Ambientales, Instituto Universitario de Investigación Marina (INMAR), Campus de Excelencia Internacional del Mar (CEIMAR), Universidad de Cádiz, Cádiz, Spain; ^4^Interdisciplinary Laboratory of Clinical Analysis INTERLAB-UMU, University of Murcia, Murcia, Spain

**Keywords:** anesthesia, clove oil, MS222, marine fish, antioxidants

## Abstract

The use of anesthesia is a common practice in aquaculture to sedate fish and mitigate handling stress. Although the employ of anesthesia is considered beneficial for fish, as it reduces stress and improves welfare, at the same time it may induce hazardous side-effects. The aim of the present study was to investigate the effects of clove oil (CO) and tricaine methanesulfonate (MS222), two of the most used anesthetics, on several oxidative stress related parameters in gilthead sea bream (*Sparus aurata*), as these types of effects of anesthetics have been seldom investigated. To assess these effects, *S. aurata* juveniles were placed in a setup of mobile water tanks and were transported during 6 h with either 2.5 mg/L CO or 5 mg/L MS222. After transport, half of the fish were sampled, whereas the remaining fish were transferred to tanks without anesthetics where they were allowed to recover for 18 h before sampling. Changes in the expression levels of several target genes related with the antioxidant response and cell-tissue repair were evaluated in the gills, liver and brain. Those transcripts included glutathione peroxidase 1 (*gpx1*), catalase (*cat*), glutathione S-transferase 3 (*gst3*), glutathione reductase (*gr*), superoxide dismutase [Zn] (*sod2*), heat shock protein-70 (*hsp70*), and metallothionein (*mt*). Antioxidant enzymatic activities glutathione S-transferase, GST; catalase, CAT; and glutathione reductase, GR, levels of non-enzymatic antioxidants (non-protein thiols – NPT), and pro-oxidative damage, assessed as lipid peroxidation (LPO), were determined in gills, liver and brain. Acetylcholinesterase activity (AChE) was determined in plasma, gills, brain, muscle and heart as an indicator of neuro-muscular alterations. In plasma, the total antioxidant capacity (TAC) and total oxidative status (TOS) were also measured. Results showed that the use of both anesthetic agents, CO and MS222, interferes with fish antioxidant status. All tested biological matrices displayed alterations in antioxidant endpoints, confirming that these substances, although minimizing the effects of transport stress, may have long term effects on fish defenses. This result is of high relevance to aquaculture considering that the oxidative stress, may increase the susceptibility to different environmental or biotic stress and different types of pathologies.

## Introduction

The use of light anesthesia or sedation on fish is a common practice in aquaculture to reduce the stress due to handling, transport, vaccination and blood sampling by depressing fish central and peripheral nervous systems. Tricaine methanesulfonate (MS222) is one of the most widely used anesthetics in fish and it is licensed for use in food fish in the United States and the European Union (FDA, 1997; European Commission, 2010; Velisek et al., 2011; Priborsky and Velisek, 2018). Clove oil (CO) has been proposed as a natural alternative and safer anesthetic, easily available and less expensive than MS222 with demonstrated efficiency in several fish species, including gilthead sea bream, *Sparus aurata* (Tort et al., 2002; Vera et al., 2010; Bodur et al., 2018). This anesthetic is a natural essential oil obtained from the clove tree (*Syzygium aromaticum*) and has as active ingredients eugenol and iso-eugenol (Javahery et al., 2012). This anesthetic has been used in several species, including *S. aurata* (Mylonas et al., 2005; Priborsky and Velisek, 2018). The iso-eugenol is also licensed for use in food fish in the European Union (European Commission, 2011), but not clove oil. The two compounds, MS222 and CO, are administrated by immersion of fish in water with the dissolved anesthetic, and thus mainly absorbed through the gills (Sneddon, 2012), biotransformed by the liver and probably the kidneys and mainly excreted through the gills and, to a lesser extent, through the urine and bile (Carter et al., 2011; Javahery et al., 2012).

The use of anesthesia is considered beneficial for fish as it reduces stress in handling procedures; however, it may induce hazardous side-effects (Ross and Ross, 2008; Priborsky and Velisek, 2018). It was previously demonstrated that MS222 induced an avoidance behavior in *Danio rerio* and *Oryzias latipes* (Readman et al., 2013, 2017), affected the natural cytotoxicity activity of head kidney leucocytes in *S. aurata* (Cuesta et al., 2003), and upregulated gill osmoregulatory genes in *Salmo salar* (Chance et al., 2018). On the other hand, CO has been shown to induce changes in some hematological parameters in *Carassius auratus* (Abdolazizi et al., 2011), enhance several skin mucosal immune parameters in *Oncorhynchus mykiss* (Soltanian et al., 2018) and induce the activity of serum and skin mucus natural hemolytic complement in *S. aurata* (Guardiola et al., 2018). However, the potential side effects of these anesthetic compounds on fish are not completely clear. These effects are species-specific and also dependent on compounds’ pharmacodynamics in the organism, which are related to biological factors and water physico-chemical parameters (Ross and Ross, 2008).

The toxicity of xenobiotics often depends on their capacity to increase cellular levels of reactive oxygen species (ROS), which can happen by activation of their synthesis or by an imbalance in the antioxidant defenses (Oliveira et al., 2013). Among antioxidant defenses, non-enzymatic (e.g., non-protein thiols – NPT), as well as enzymatic responses (e.g., glutathione reductase – GR; glutathione *S*-transferases – GST and catalase, CAT) are frequently assessed as biomarkers of xenobiotic mediated oxidative stress (Oliveira et al., 2013). Overwhelmed antioxidant defenses may result in oxidative stress, which may be responsible for pernicious effects like inactivation of enzymes and peroxidative damage (e.g., lipid peroxidation – LPO). Metallothioneins (MT) are known to be over-expressed in organisms from environments with high metal concentrations (Viarengo et al., 1999). Nevertheless, they may also be used as biomarkers of a general stress response to environmental contaminants (Oliveira et al., 2009). It has been demonstrated that the MT gene promoter region contains not only genetic elements responsive to metals, but also sequences thought to be involved in oxidative stress response (Oliveira et al., 2009). Heat shock proteins (HSP) are also markers of cellular stress. Thus, both MT and HSP can also be used as biomarkers of a general cellular stress response to drugs/xenobiotics. In blood plasma, the total antioxidant capacity (TAC), that reflects the combined action of different antioxidants and the total oxidant status (TOS), that measures the different oxidant species present, have been proposed as valuable endpoints to assess the oxidative status in fishes after exposure to chemicals (Teles et al., 2016).

The aim of the present study was to investigate the potential side effects of MS222 and CO on the oxidative status (in gills, plasma, liver, and brain), neurotransmission (in gills, plasma, liver, muscle, and heart), immune function (in plasma) and cellular stress (in gills, liver, and brain) in gilthead sea bream (*S. aurata*) after a simulated transportation and a recovery period. Sedation doses of anesthetics are proposed to reduce stress during transport (Zahl et al., 2012; Vanderzwalmen et al., 2018), and *S. aurata* is an important aquaculture species with a high commercial importance. The selection of tissues was based on functions and anatomic locations. Gills were selected as they are a multifunctional organ with essential osmoregulatory, respiratory and immunologic roles (Evans et al., 2005) in close contact with the external medium and waterborne environmental substances (Oliveira et al., 2009); the liver is a major organ in terms of body weight, with relevant functions in energetics, intermediary metabolism, and storage of substances. The brain is the target organ of anesthetic agent’s action in fish, and thus a potential target of side effects that may include oxidative stress; and, finally, blood has an important role in the transfer of substances absorbed through the gills, skin and gut, and provides information on the overall health status of the animal. In this regard, these biological matrices were considered candidates for the detection of early warning signs of possible harmful effects of MS222 and CO used for the transport of *S. aurata* individuals.

## Materials and Methods

### Fish Husbandry and Experimental Design

Juveniles of *S. aurata* were obtained from Servicios Centrales de Investigación en Cultivos Marinos (SCI-CM, CASEM, University of Cádiz, Puerto Real, Cádiz, Spain; Spanish Operational Code REGA ES11028000312). Immature gilthead sea bream (*S. aurata*) juveniles (*n* = 72, 42.7 ± 6.8 g body mass, mean ± SD) were transferred to the facilities from the Department of Biology at the Faculty of Marine and Environmental Sciences (CASEM, University of Cádiz, Puerto Real, Cádiz, Spain) and acclimated to laboratory conditions for 7 days in four flow-through 500 L seawater tanks (approximate stocking density: 3.5 kg m^−3^), under stable conditions of salinity (38), temperature (19°C), and natural photoperiod for this season and latitude (May 2015; 13:11 h, light:dark; 36°31′45″ N, 6°11′31″ W). Animals were fed two times daily (1% of tank biomass per day) with commercial pellets for *S. aurata* (Skretting España S.A., Spain).

For the transport simulation, fish were randomly placed into a mobile setup of nine 15 L aquaria, and distributed in three different experimental groups in triplicate. Then, *S. aurata* juveniles were transported for 6 h with either 2.5 mg of CO L-1 (extracted from cloves of Eugenia spp., Sigma-Aldrich C8392) or 5 mg of MS222 L-1 (Sigma-Aldrich E10521), plus a control group without anesthetics.

Tested concentrations are related to anesthesia induction characterization previously done in gilthead sea bream with both anesthetics. The selected doses of the anesthetics were half of the lowest concentration that induced a light sedation in a previous characterization performed (data not shown). Higher concentrations were not considered in this experimental design to avoid the induction of deeper sedation or anesthesia stages during the transport of fish.

No changes in behavioral or clinical effects due to anesthetics administration were determined during the transport or recovery period. All aquaria were fully oxygen-saturated with air stones. Animals were fasted for 24 h before the assay. To simulate transport conditions, every 20 min the mobile setup of aquaria was displaced for 5 min (mimicking noise and vibrational disturbances due to shaking) followed by 15 min of resting. After 6 h half of the animals were euthanized and sampled (*n* = 12 per experimental group, *n* = 4 animals per tank), whereas the remaining fish were transferred to similar clean-water aquaria to allow them to recover for 18 h, and then euthanized and sampled as well. Sampled animals were netted and deeply anesthetized with 2-phenoxyethanol (1 mL L^−1^, Sigma-Aldrich 77699) for blood collection. 2-phenoxyethanol was selected due to its low time to induce deep anesthesia (less than 1 min) and to standardize the possible side-effects among groups (control, MS222 and CO). After blood collection (in less than 3 min since capture), fish were euthanized by spinal cord sectioning. Plasma was isolated by centrifugation (3 min, 10000 × *g*, 4°C), and frozen in liquid nitrogen. Gills, liver and brain were excised, flash frozen in liquid nitrogen and posteriorly used for gene expression analysis and biochemical endpoints assessment. The experiment complied with the Guiding Principles for Biomedical Research Involving Animals (EU2010/63), the guidelines of the Spanish laws (law 32/2007 and RD 53/2013), and authorized by the Ethical Committee of the Universidad de Cádiz (Spain) for the use of laboratory animals and the Ethical Committee from the Andalusian Government (Junta de Andalucía reference number 28-04-15-241).

### RNA Isolation, Retrotranscription, and Real-Time Quantitative PCR

Total RNA was extracted from the selected tissues of control and exposed fish using Tri Reagent^^®^^ (Sigma-Aldrich) and following manufacturer’s recommendations. RNA quantification was done using a NanoDrop Spectrophotometer (Thermo Fisher Scientific, United States) and RNA quality checked with Experion, using the Experion Standard Sens RNA chip (Bio-Rad Laboratories, United States). Reverse transcription was performed using 1 μg of the total RNA using the iScript^TM^ cDNA synthesis kit (Bio-Rad, United States) according to the manufacturer’s instructions. Efficiency of the amplification was determined for each primer pair using serial fivefold dilutions of pooled cDNA and calculated as *E* = 10 (−1/s), where s is the slope generated from the serial dilutions. RT-qPCR was run in a Bio-Rad CFX384 Real-Time PCR Detection System (Bio-Rad, United States). Reactions were done using iTaqTM Universal SYBR^^®^^ Green Supermix (Bio-Rad, United States) according to the manufacturer’s instructions. Briefly, 1 cycle at 95°C for 5 min, 40 cycles at 95°C for 30 s, 60°C for 30 s, and 72°C for 30 s were run; samples were performed in triplicates. Expression data, obtained from three independent biological replicates, were used to calculate the threshold cycle (Ct) value. After checking for primers efficiency, RT-qPCR analysis of all the individual samples was determined following the same protocol described above. NormFinder was used to identify the most appropriate housekeeping gene among the tested 3: elongation factor-1α (*ef1α*), α-tubulin (*tub*), and β-actin (*act*). Stability values of the individual candidate housekeeping genes were: 0.018 for ef1α, 0.022 for act, and 0.028 for tub in the gills; 0.017 for *ef1α* and *act*, and 0.018 for tub in the liver; 0.022 for tub, 0.038 for act, and 0.052 for ef1α in the brain. The best combination of two-genes was act + tub with a stability value of 0.017 and 0.010 for the gills ant the liver, respectively. For the brain, the best combination of two-genes was ef1α + act with a stability value of 0.029. The best combination of two genes for each organ was used for normalization. Normalized gene expression calculated with the ΔΔCt method (Livak and Schmittgen, 2001). Primers information is given in Table 1.

**Table 1:** Sequences and efficiencies of primers used for quantitative real-time PCR analysis.

Gene name	Acronym	GenBank ID	Forward	Reverse
Elongation factor-1α	*ef1*α	AF184170	CCCGCCTCTGTTGCCTTCG	CAGCAGTGTGGTTCCGTTAGC
α-Tubulin	*tub*	AY326430	AAGATGTGAACTCCGCCATC	CTGGTAGTTGATGCCCACCT
β-Actin	*actin*	X89920	TCCTGCGGAATCCATGAGA	GACGTCGCACTTCATGATGCT
Glyceraldehyde 3-phosphate dehydrogenase	*gapdh*	DQ641630	TGCCCAGTACGTTGTTGAGTCCAC	CAGACCCTCAATGATGCCGAAGTT
Catalase	*cat*	JQ308823	TGGTCGAGAACTTGAAGGCTGTC	AGGACGCAGAAATGGCAGAGG
Superoxide dismutase (Mn)	*sod2*	JQ308833	CCTGACCTGACCTACGACTATGG	AGTGCCTCCTGATAT TTCTCCTCTG
Glutathione peroxidase 1	*gpx1*	DQ524992	GAAGGTGGATGTGAATGGAAAAGATG	CTGACGGGACTCCAAATGATGG
Glutathione reductase	*gr*	AJ937873	TGTTCAGCCACCCACCCATCGG	GCGTGATACATCGGAGTGAATGAAGTCTTG
Glutathione-S-transferase 3	*gst3*	JQ308828	CCAGATGATCAGTACGTGAAGACCGTC	CTGCTGATGTGAGGAATGTACCGTAAC
Metallothionein	*mt*	U93206	CTCTAAGACTGGAACCTG	GGGCAGCATGAGCAGCAG
Heat shock protein 70	*hsp70*	EU805481	AATGTTCTGCGCATCATCAA	GCCTCCACCAAGATCAAAGA

### Sample Preparation for Biochemical Analyses

The samples of tissues for biochemical analysis were homogenized in potassium phosphate buffer (0.1 M, pH 7.4). Aliquots of the homogenate were separated for non-protein thiols (NPT), lipid peroxidation (LPO) assessment and post-mitochondrial fraction (PMS) isolation (30 min at 15,000 *g*, 4°C).

### Biochemical Analyses

The protein content of the homogenates and PMS fractions was determined. Protein content was determined by the Bradford (1976) method adapted to microplate. Prior to enzymatic analysis, the content of protein in the samples was normalized to 0.9 mg/mL. After enzymatic determinations, the protein concentration in each sample was quantified again and the measured value used to express the enzymatic activities per protein (Oliveira et al., 2012). Cholinesterase activity was determined in the gills, brain and heart was determined according to Ellman et al. (1961) method adapted to microplate, at 25°C. In the blood, AChE was determined according to Tecles and Cerón (2001) adapted for an automated analyzer (Olympus AU600; Olympus Diagnostica GmbH, Hamburg, Germany). The results were expressed as nmol of thiocholine formed per minute per mg of protein. Non-protein thiols content was estimated generally following the procedure described by Oliveira et al. (2010). Results were expressed as millimoles per milligram of protein. Catalase activity was determined by the method of Claiborne (1985) adapted to microplate. Absorbance was recorded spectrophotometrically at 240 nm (25°C) and enzyme activity expressed as micromoles H_2_O_2_ consumed per minute per milligram protein. Glutathione *S*-transferase activity was measured according to Habig et al. (1974) adapted to microplate. Absorbance was recorded at 340 nm (25°C) and expressed as nmol CDNB conjugate formed per minute per mg of protein. Glutathione reductase activity was assayed by the method of Carlberg and Mannervik (1975) adapted to microplate. Enzyme activity was quantified by measuring NADPH decrease at 340 nm (25°C) and expressed as nmol NADPH oxidized/min/mg protein. TAC was assessed in the plasma as described by Erel (2004), by measuring the 2,2′-azinobis-(3-ethylbenzothiazoline-6-sulfonate) decolorization by antioxidants at 660 nm. The activity was expressed as mmol/L. TOS was measured in plasma as described by Erel (2005). The method is based on the reaction that the ferric ion makes a colored complex with xylenol orange in an acidic medium. The color intensity, which was measured spectrophotometrically at 560 nm (Olympus Diagnostica, GmbH) using 800 nm as the reference, is related to the total amount of oxidant molecules present in the sample. The assay was calibrated with hydrogen peroxide and the results were expressed in terms of micromole hydrogen peroxide equivalent per liter (μmolH_2_O_2_Equiv/L). Lipid peroxidation levels were determined according to the protocols described by Oliveira et al. (2009). Absorbance was measured at 535 nm and LPO was expressed as nmol of thiobarbituric acid reactive substances (TBARS) formed per mg of protein.

### Data and Statistical Analysis

Statistical analysis was done using the IBM SPSS Statistics 22 software package. One-way ANOVA was performed followed by the Dunnett’s test to signal significant differences between treated and control groups. Statistically significant differences between treated groups and their respective controls groups are denoted with asterisks. One asterisk indicates *p* < 0.05, two asterisks indicate *p* < 0.001.

## Results

### Molecular Responses

**FIGURE 1:**
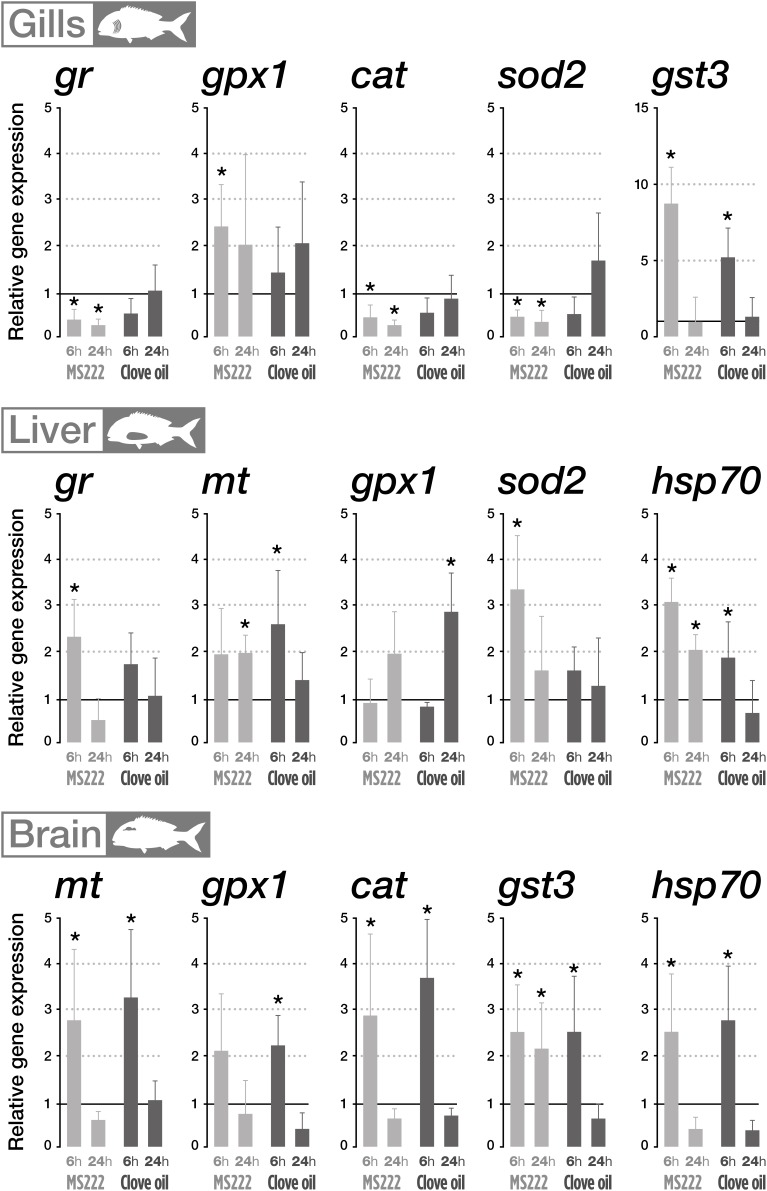
Relative mRNA levels of target genes measured on gills, liver and brain of *S. aurata* sedated with tricaine methanesulfonate (MS222) or clove oil (CO) after 6 h transportation and 18 h recovery. The horizontal line originating at *y* = 1 denote the control group against which the expression was normalized. Bars represent the mean value of six fish with standard error. An asterisk indicates statistical significances compared to the control group (*p* < 0.05). Only parameters that denote statistically significant changes are presented.

In gills, organisms treated with MS222 increased *gpx1* and *gst3* mRNA levels significantly after 6 h transportation, with mRNA levels returning to control values after 18 h recovery (Figure 1). However, *gr*, *cat*, and *sod2* expression levels significantly decreased after transportation and recovery period. Gill *mt* and *hsp70* mRNA levels were unaltered (data not shown). For CO treated fish, the gill’s mRNA levels of *gst3* enhanced after transportation, returning to control values after recovery. However, the rest of transcripts assessed (*gpx1*, *cat*, *gr*, *mt*, and *hsp70*) didn’t present any change.

Fish exposed to MS222 enhanced hepatic *sod2* and *gr* mRNA levels after 6 h of transportation, returning to control levels after 18 h of recovery; while no alterations were observed in mRNA levels of *gpx1* (Figure 1) as well as *cat* and *gst3* (data not shown). Respect to the assessed cellular stress indicators, although no significant effects were detected after transportation, MS222 stimulated significantly *mt* mRNA levels after recovery period. Finally, *hsp70* mRNA values were significantly higher than controls at both sampling periods. Concerning CO treated animals, a significant increase in *gpx1* mRNA levels was found after recovery period despite the lack of effects after transportation. All the other antioxidant related transcripts presented no significant differences with respect to control at both sampling points. However, for the cellular stress indicators (*mt* and *hsp70*), CO enhanced significantly hepatic mRNA expression after transportation but expression returned to control levels after recovery period.

In the brain, the use of MS222 during transportation increased mRNA levels of *mt*, *cat*, and *hsp70* after 6 h but returned to control levels after recovery period (Figure 1). Expression levels for *gst3* were significantly higher than controls after 6 and 24 h; while the expression of *gpx1* and *gr* displayed no significant differences from control throughout the experiment. CO, induced a similar pattern of change in expression levels of mt, *gpx1*, *cat*, gst3, and *hsp70*, with a significantly enhancement after transportation and returning to control levels after recovery period (Figure 1). The expression of *gr* in the brain was unaltered throughout the experimental assay (data not shown).

### Biochemical Responses

**FIGURE 2:**
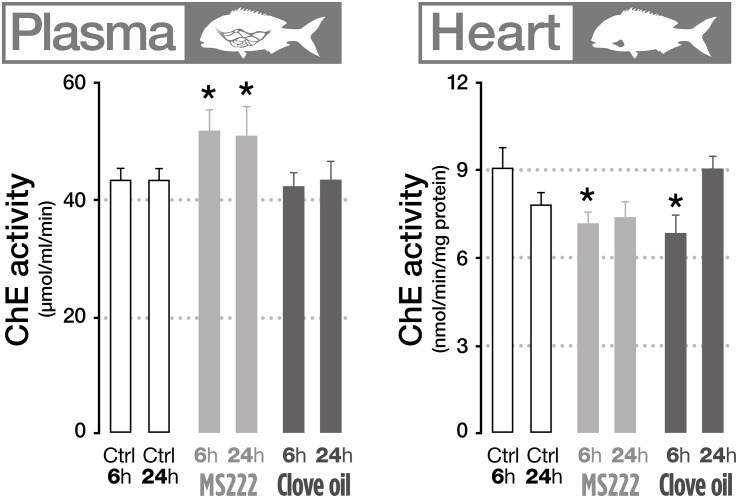
Cholinesterase activity in plasma and heart of *S. aurata* sedated with tricaine methanesulfonate (MS222) or clove oil (CO) after 6 h transportation and 18 h recovery. Bars represent the mean value of six fish with standard error. An asterisk indicates statistical significances compared to the control group (*p* < 0.05). Only parameters that denote statistically significant changes are presented.

The effects of MS222 and CO on neurotransmission indicators were assessed through the measurement of AChE activity on different biological matrices (gills, plasma, brain, muscle, and heart). Results showed that fish treated with MS222 significantly increased AChE activity in plasma both after 6 h transportation and after recovery period (Figure 2). However, in the heart, AChE was significantly inhibited after transportation, returning to control levels after 24 h recovery. Fish treated with CO only displayed significant differences to control in the heart, with a decreased activity after transportation (Figure 2).

**FIGURE 3:**
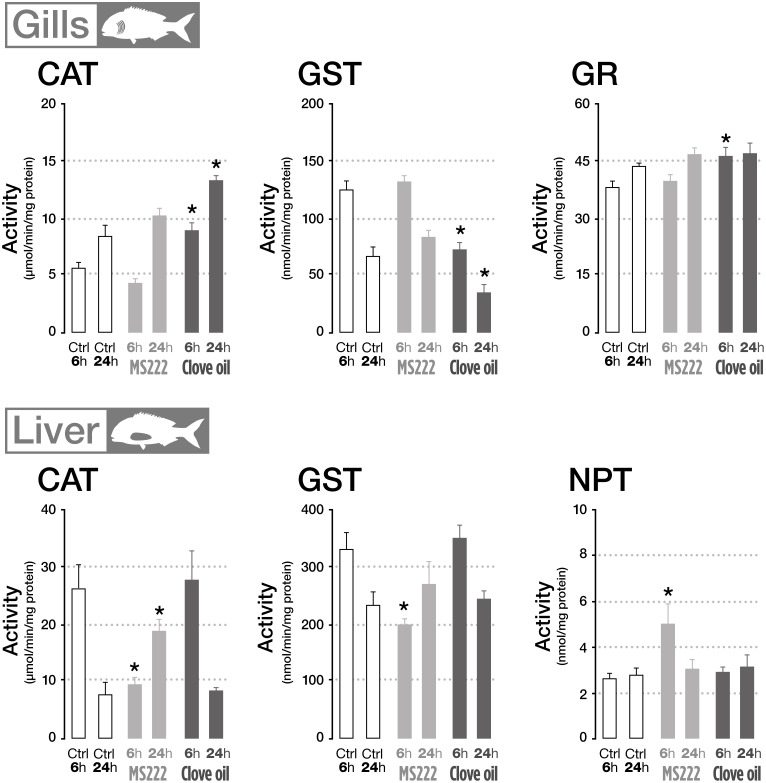
Oxidative stress related parameters in the gills and liver of *Sparus aurata* sedated with tricaine methanesulfonate (MS222) or clove oil (CO) after 6 h transportation and 18 h recovery. An asterisk indicates statistical significances compared to the control group (*p* < 0.05). Only parameters that denote statistically significant changes are presented.

In terms of oxidative stress related parameters, plasma TAC and TOS levels in fish exposed to MS222 and CO were not significantly affected, when compared to their respective controls (data not shown). Gills enzymatic antioxidant defenses were not responsive to MS222 treatment, whereas CO treated organisms significantly increased CAT activity and decreased GST, at both sampling periods; while GR was significantly enhanced after transportation (Figure 3).

Considering hepatic enzymatic defenses, CAT and GST activities in fish exposed to MS222 displayed a significant decrease after 6 h transportation returning to control levels after recovery. No significant differences were found between controls and CO treated fish in terms of hepatic enzymatic antioxidant defenses (Figure 3). The assessed antioxidant defenses were not responsive to the tested anesthetics in the brain (data not shown). No significant differences between control and treated groups were found in the tested organs in terms of peroxidative damage (data not shown). Respect to non-enzymatic antioxidant defenses, assessed in this study through the quantification of NPT levels, differences to control situation were only found in the liver of fish treated with MS222 after 6 h transportation, returning to basal levels after recovery (Figure 3).

## Discussion

This study aimed to evaluate potential oxidative side effects of the use of MS222 and CO anesthetics in a simulated transportation of fish. These substances are being used in practice to reduce risk of stress-induced problems such as injuries, feeding and immune depression that may induce relevant physiological alterations in fish and compromise its development, overall health and welfare (Ross and Ross, 2008; Priborsky and Velisek, 2018). In addition, slight sedation during transport has been proved to reduce the metabolic rate of fish, and consequently oxygen consumption and generation of waste products, improving water quality management (Zahl et al., 2012; Vanderzwalmen et al., 2018). Despite the recognized applications of anesthetics, the side effects of anesthesia are unknown in many fish species (Bowker et al., 2015), being responses to these chemicals species- and anesthetic-specific (Readman et al., 2017). Thus, it becomes highly relevant to characterize the potential side effects of MS222 and CO to aquaculture fish-species, like *S. aurata*, where the use of anesthetics is a possible solution to improve animal welfare (Ashley, 2007). Our study confirmed that the use of anesthetics may modulate fish antioxidant responses. This is particularly relevant considering that here may be energetic costs to re-establish the oxidative status and that oxidative stress has been associated with an increased susceptibility to different abiotic and biotic stressors, development of different pathologies and constraint on growth.

The susceptibility of a given organ to a substance is modulated by (i) the different predisposition to accumulate the substance, (ii) its characteristic antioxidants basal levels, (iii) its adaptation capacity and consequent antioxidant activation, and (iv) the metabolic rate, thus increasing the potential to produce ROS and challenge the respective defenses (Oliveira et al., 2008). The assessed mRNA levels revealed that gills were more responsive to MS222 than to CO.

The observed increased expression of *gpx1* and *gst3* suggests production of reactive species (e.g., hydroperoxides) and activation of phase II biotransformation as well as an oxidative status that could impair gills’ *sod2*, *cat*, and *gr* mRNA levels. However, hepatic expression levels of the assessed target genes suggest that liver was less responsive to MS222 than gills.

Only *sod2*, *cat*, and *hsp70* were responsive after 6 h transportation, increasing mRNA levels. However, after the recovery period, only *hsp70* remained different from controls. Despite not being responsive during transportation, *mt* mRNA levels appeared/were enhanced after recovery period. These responses support the idea that the liver presents basal antioxidant levels capable of protecting the cells from MS222 induced damage, despite the occurrence of cellular stress, as suggested by the *mt* and *hsp70* altered levels after recovery. Supporting the idea of increased ROS levels is the induction of *mt* mRNA levels, which have been reported to respond to non-metal compounds able to cause oxidative stress in fish cells, despite its upregulation by metals (Oliveira et al., 2009). Despite the lack of inhibition of the assessed antioxidants’ mRNA levels, *hsp70* mRNA expression after 6 h transportation suggests the presence of stress. Contrary to the observed in the gills, brain *cat* expression levels increased after transportation that could indicate lower susceptibility, likely associated with higher basal levels of antioxidants and higher responsiveness than gills. The mRNA levels of *gst3* in the brain were, like in the gills, significantly increased after 6 h transportation but, for this organ, mRNA levels remained higher than controls even after the recovery period, suggesting the formation of hydroperoxides. The *mt* and *hsp70* levels in the brain were also responsive to MS222 associated cellular stress. Concerning CO, gills’ *gst3* expression values displayed a pattern of response similar to MS222. However, the mRNA levels of the other assessed endpoints were not altered when compared to controls suggesting a lower ability of CO to induce ROS. Nonetheless, changes in hepatic *mt* and *hsp70* expression suggest the existence of cellular stress that, after 24 h, disappears. However, *gpx1* was increased in the recovery period, probably due to an increased in ROS production. The brain appears more sensitive to CO than to MS222, with increased expression of *cat*, *gst3*, *mt*, and *hsp70* after 6 h that, after recovery return to control levels.

Considering the molecular responses, two of the most commonly assessed enzymatic activities, GST and CAT, were determined in the three organs, to understand the molecular responses. MS222 displayed no significant effects on gills’ GST activity, despite the increased expression of *gst3* and, in the liver, the 6 h exposure caused a decreased significantly enzymatic activity. The brain GST activity was also not affected by MS222 despite the observed increased expression of *gst3*. CAT activity in the gills and brain was not significantly altered by MSS222, unlike hepatic GST activity that was inhibited after 6 h exposure despite the non-significant decrease in *gst3* mRNA expression. Concerning CAT activity, only the liver was sensitive to the treatment, with a significant decrease after 6 h and an increase after the recovery period. Previous studies have reported the ability of MS222 to decrease CAT activity in the erythrocytes of the marine fish *Dicentrarchus labrax* (Gabryelak et al., 1989). The non-enzymatic antioxidants in the liver and brain, show that liver was responsive to the MS222 6 h treatment, increasing the antioxidant defenses. With respect to CO, the biochemical endpoints suggest an ability to inhibit GST activity and induce the activity of CAT in the gills, effects that remain even after the recovery period and were not associated with effects on non-enzymatic antioxidants. In terms of peroxidative damage, the tested treatments revealed no effects. The TOS and TAC levels in the plasma, that have been proposed as a markers of the oxidative status in fish, showed that the anesthetics displayed no significant differences to control.

Overall, the data show that, under the present experimental design, anesthetics are able to affect biochemical endpoints in gills, liver and brain without significant consequences to the health of the tested organisms. Based on the molecular endpoints, CO appears to induce less effects or effects from which the fish are able to more easily recover than MS222. This could be expected based on the reports in different *in vitro* assays that show that CO can be an antioxidant, having reducing power, superoxide anion radical scavenging, hydrogen peroxide scavenging and metal chelating activities (Gülçin et al., 2012). The ability of MSS22 to affect antioxidant defenses such as peroxide metabolism enzymes (Gabryelak et al., 1989) and thus to compromise their use as biomarkers has been previously reported. Considering the effects on neurotransmission, data suggest that exposure to MS222 and CO may affect neurotransmission as significant decreases were found in the heart, despite the lack of effects observed for the other tissues. Furthermore, the increased levels of AChE found in the plasma of fish treated with MS222 may suggest pernicious effects of this anesthetic as previous studies have associated increases in AChE activity with ongoing apoptotic processes in the organism (Zhang et al., 2002, 2012) and production of free radicals and oxidative stress (Ferreira et al., 2012).

In conclusion, our results indicate that the use of both anesthetic agents, CO and MS222, interfere with fish antioxidant status. All tested biological matrices displayed alterations in antioxidant endpoints, confirming that these substances, although minimizing the effects of transport stress, may have long term effects on fish defenses. These results are of high relevance to aquaculture considering that an altered oxidative stress, induced by use of these anesthetics as stress-reducing agent during transport, may increase the susceptibility to different environmental or biotic stress and promote different types of pathologies.

## Ethics Statement

The experiment complied with the Guiding Principles for Biomedical Research Involving Animals (EU2010/63), the guidelines of the Spanish laws (law 32/2007 and RD 53/2013), and authorized by the Ethical Committee of the Universidad de Cádiz (Spain) for the use of laboratory animals and the Ethical Committee from the Andalusian Government (Junta de Andalucía reference number 28-04-15-241).

## Author Contributions

JM and IJ-C designed the experiments and performed the experimental setup. IJ-C, MT, and MO conducted the experimental procedures. MT, MO, LF-M, and AT did the laboratorial analysis and analyzed the results. MT and MO wrote the manuscript. LT and JM revised the manuscript. All authors read and approved the manuscript for publication.

## Conflict of Interest Statement

The authors declare that the research was conducted in the absence of any commercial or financial relationships that could be construed as a potential conflict of interest.
